# Genome Sequences of 15 *Gardnerella vaginalis* Strains Isolated from the Vaginas of Women with and without Bacterial Vaginosis

**DOI:** 10.1128/genomeA.00879-16

**Published:** 2016-09-29

**Authors:** Lloyd S. Robinson, Justin Perry, Sai Lek, Aye Wollam, Erica Sodergren, George Weinstock, Warren G. Lewis, Amanda L. Lewis

**Affiliations:** a Department of Molecular Microbiology, Washington University School of Medicine, St. Louis, Missouri, USA; b Center for Women’s Infectious Disease Research, McDonnell Genome Institute, Washington University School of Medicine, St. Louis, Missouri, USA; c Department of Medicine, Washington University School of Medicine, St. Louis, Missouri, USA; d Department of Obstetrics and Gynecology, Washington University School of Medicine, St. Louis, Missouri, USA

## Abstract

*Gardnerella vaginalis* is a predominant species in bacterial vaginosis, a dysbiosis of the vagina that is associated with adverse health outcomes, including preterm birth. Here, we present the draft genome sequences of 15 *Gardnerella vaginalis* strains (now available through BEI Resources) isolated from women with and without bacterial vaginosis.

## GENOME ANNOUNCEMENT

Bacterial vaginosis (BV) is a common condition characterized by low levels of “healthy” vaginal lactobacilli and overgrowth of diverse anaerobes (1, 2). Women with BV have higher risks of many adverse health outcomes, including HIV transmission, intrauterine infections, and preterm birth (3–7). Unfortunately, the condition is highly recurrent; most women experience another episode of BV within months of treatment (8, 9). *Gardnerella vaginalis* is a facultative anaerobe in the family *Bifidobacteriaceae* and is one of the dominant species in BV (10). Recent studies demonstrated that *G. vaginalis* (strain JCP8151B) was sufficient to cause several features of BV in a mouse vaginal infection model (11, 12). To support further studies aimed at understanding the role of *G. vaginalis* in BV, we sequenced the genomes of 15 *G. vaginalis* strains (including JCP8151B) that were isolated from the vaginal swabs from 12 women enrolled in the institutional review board (IRB)-approved Washington University Contraceptive CHOICE Project (13).

Strains were isolated by plating vaginal swabs on *Gardnerella* semiselective medium, as described previously (12). After 24 to 48 h of growth at 37°C in an anaerobic chamber, pinpoint colonies were subcultured and tested by PCR using *Gardnerella*-specific primers (14). Full-length 16S rRNA gene sequences confirmed their identity. All strains were derived from different women, with the exception of three strain pairs sharing the same numerical identifier (i.e., JCP8151A and JCP8151B, JCP8017A and JCP8017B, and JCP8481A and JCP8481B).

For genome sequencing, strains were grown overnight in NYCIII broth, and DNA was isolated with the Wizard genomic DNA purification kit (Promega). Shotgun libraries were generated and sequenced with Illumina sequencing technology. Contigs were assembled with Velvet 1.1.06. GeneMark and Glimmer3 (15, 16) were used to predict coding regions, and the remaining sequences were examined for homology to sequences in the NCBI nonredundant protein database. Putative protein functions were predicted with a suite of programs, including KEGG (17), PSORTb (18), and InterProScan (19). tRNAscan-SE was used to identify tRNAs (20), and additional noncoding RNA genes were determined with RNAmmer (21) and Rfam (22).

The average genome coverage of the sequenced strains was 150×. The average genome size was 1.6 Mbp. The strains contain, on average, 2,800 protein-coding genes, 15 rRNA genes, and 92 tRNA genes, and have a G+C content of 42.5%.

### Accession number(s).

The draft genome sequences for the 15 *G. vaginalis* strains have been deposited in GenBank under the accession numbers listed in Table 1. The sequences described in this paper are the first versions. We have also made 14 of these strains available to the research community by depositing them with the Biodefense and Emerging Infections (BEI) Research Resource Repository (see BEI numbers in Table 1).

**TABLE 1:** GenBank and BEI accession numbers of the sequenced *G. vaginalis* strains

Strain/BEI no.	BEI accession no.	Nucleotide accession no.
JCP7275	HM-1105	ATJS00000000
JCP7276	HM-1106	ATJR00000000
JCP7659	HM-1107	ATJQ00000000
JCP7672	HM-1108	ATJP00000000
JCP7719	HM-1109	ATJO00000000
JCP8017A	HM-1110	ATJN00000000
JCP8017B	HM-1111	ATJM00000000
JCP8066	HM-1112	ATJL00000000
JCP8070	HM-1113	ATJK00000000
JCP8108	HM-1114	ATJJ00000000
JCP8151A	HM-1115	ATJI00000000
JCP8151B	HM-1116	ATJH00000000
JCP8481A	NA^a^	ATJG00000000
JCP8481B	HM-1118	ATJF00000000
JCP8522	HM-1119	ATJE00000000

a NA, not available.
